# Entorhinal theta-frequency input to the dentate gyrus trisynaptically evokes hippocampal CA1 LTP

**DOI:** 10.3389/fncir.2012.00064

**Published:** 2012-09-12

**Authors:** Jens Stepan, Julien Dine, Thomas Fenzl, Stephanie A. Polta, Gregor von Wolff, Carsten T. Wotjak, Matthias Eder

**Affiliations:** ^1^Research Group Neuronal Network Dynamics, Max Planck Institute of PsychiatryMunich, Germany; ^2^Research Group Neuronal Plasticity, Max Planck Institute of PsychiatryMunich, Germany

**Keywords:** LTP, hippocampus, theta, trisynaptic circuit, entorhinal cortex, voltage-sensitive dye, caffeine, corticosterone

## Abstract

There exists substantial evidence that some forms of explicit learning in mammals require long-term potentiation (LTP) at hippocampal CA3-CA1 synapses. While CA1 LTP has been well characterized at the monosynaptic level, it still remains unclear how the afferent systems to the hippocampus can initiate formation of this neuroplastic phenomenon. Using voltage-sensitive dye imaging (VSDI) in a mouse brain slice preparation, we show that evoked entorhinal cortical (EC) theta-frequency input to the dentate gyrus highly effectively generates waves of neuronal activity which propagate through the entire trisynaptic circuit of the hippocampus (“HTC-Waves”). This flow of activity, which we also demonstrate *in vivo*, critically depends on frequency facilitation of mossy fiber to CA3 synaptic transmission. The HTC-Waves are rapidly boosted by the cognitive enhancer caffeine (5 μM) and the stress hormone corticosterone (100 nM). They precisely follow the rhythm of the EC input, involve high-frequency firing (>100 Hz) of CA3 pyramidal neurons, and induce NMDA receptor-dependent CA1 LTP within a few seconds. Our study provides the first experimental evidence that synchronous theta-rhythmical spiking of EC stellate cells, as occurring during EC theta oscillations, has the capacity to drive induction of CA1 LTP via the hippocampal trisynaptic pathway. Moreover, we present data pointing to a basic filter mechanism of the hippocampus regarding EC inputs and describe a methodology to reveal alterations in the “input–output relationship” of the hippocampal trisynaptic circuit.

## Introduction

Yet for many years, the phenomenon of long-term potentiation (LTP) at hippocampal CA3-CA1 synapses is intensively used as an experimental model for studying cellular underpinnings of learning in mammals (Bliss and Collingridge, 1993; Malinow, 2003; Malenka and Bear, 2004; Henneberger et al., 2010). Different lines of evidence corroborate that CA1 LTP occurs also naturally in the brain and, at least in several mammalian species, is essential for the acquisition of spatial and episodic memories (Morris et al., 1986; Zola-Morgan et al., 1986; Tsien et al., 1996; Burgess et al., 2002; Gruart et al., 2006; Whitlock et al., 2006). Multitudinous studies have been performed on CA1 LTP induced by high-frequency stimulation of CA3-CA1 projections (i.e., axons of CA3 pyramidal neurons). This work yielded a sophisticated understanding of induction and expression mechanisms underlying long-term plasticity at glutamatergic synapses (Bliss and Collingridge, 1993; Malinow, 2003; Malenka and Bear, 2004; Henneberger et al., 2010). In contrast, it remains largely unknown how the brain systems afferent to the hippocampus can initiate formation of CA1 LTP.

Synaptic input from the entorhinal cortex to the dentate gyrus (in the following termed “EC/DG-input”) provides the hippocampus with polymodal sensory information (Andersen et al., 2007; Neves et al., 2008). EC/DG-input, which is predominantly generated by layer II stellate cells and conveyed by perforant path (PP) fibers, activates DG granule cells (Andersen et al., 2007; Neves et al., 2008). The axon terminals of DG granule cells (mossy fiber boutons) give rise to the most prominent non-commissural/associational excitatory innervation of CA3 pyramidal neurons (Nicoll and Schmitz, 2005; Andersen et al., 2007; Neves et al., 2008). These facts suggest that EC/DG-input acts as a major “extrinsic driving force” for natural formation of CA1 LTP.

The induction of spatial and episodic memories is often associated with theta (3–8 Hz) activity in the entorhinal-hippocampal system (Winson, 1978; Mitchell et al., 1982; Buzsáki, 2002; Rutishauser et al., 2010). We therefore hypothesized that theta-rhythmical EC/DG-input might possess the capability to generate hippocampal network dynamics which can lead to formation of CA1 LTP. Here we tested this hypothesis by applying voltage-sensitive dye imaging (VSDI) and classical electrophysiological techniques to a mouse brain slice preparation. We employed VSDI since it allows the analysis of neuronal activity on a millisecond time scale, with micrometer-range spatial resolution and, most importantly, a scope spanning the entire brain circuits under study (Iijima et al., 1996; Grinvald and Hildesheim, 2004; Airan et al., 2007; Refojo et al., 2011; von Wolff et al., 2011).

## Results

**Figure 1:**
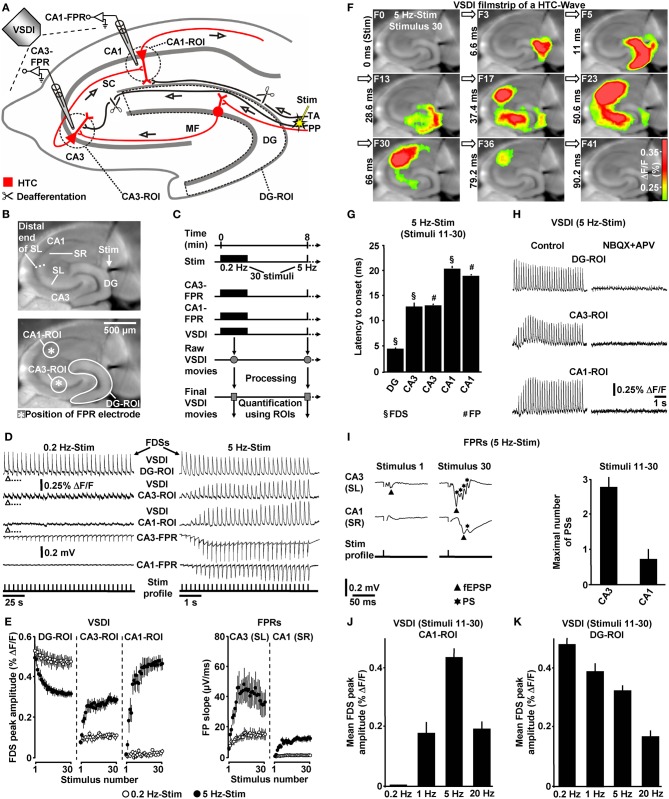
**Theta-rhythmical (5 Hz) EC/DG-input highly effectively generates neuronal activity flow through the hippocampus. (A–C)** Experimental arrangement and protocol used for the investigations shown in **(D–K)**. The experiments with 1 Hz EC/DG-input (*N* = 9 slices/6 mice) and 20 Hz EC/DG-input (*N* = 11 slices/6 mice) shown in **(J** and **K)** were performed in separate sets of slices. **(B,D,F,I)** Illustration and outcome of a representative experiment. Open triangles in **(D)** symbolize an interruption of VSDI for 4.34 s. **(E)** Quantification of neuronal activities in hippocampal subregions (*N* = 9 slices/5 mice). **(G,I)** Characteristics of the 5 Hz neuronal activities depicted and quantified in **(D–F)**. **(H)** Bath application of the AMPA/kainate receptor blocker NBQX (5 μM) and the NMDA receptor antagonist APV (50 μM) to slices fully inhibited neuronal activity in the hippocampus. **(J,K)** Quantification of CA1 and DG neuronal activities resulting from 0.2, 1, 5, and 20 Hz EC/DG-input. **(D,I)** Stimulus artifacts in FPR traces were truncated for clarity. Abbreviations: Δ*F/F*, fractional change in fluorescence; F, imaging frame (number and time specification relative to stimulation pulse); FP(R), field potential (recording); HTC, hippocampal trisynaptic circuit; MF, mossy fiber; PS, population spike; ROI, region of interest; SC, Schaffer collateral; SL, stratum lucidum; SR, stratum radiatum; Stim, extracellular electrical stimulation; TA, temporoammonic pathway.

We evoked EC/DG-input by electrical stimulation of the PP (Figures 1A,B). EC/DG-input thus resulted from synchronous spike activity in PP fibers. To ensure that hippocampal activity solely arises from EC/DG-input, PP fibers which directly innervate area CA3 and temporoammonic projections were functionally inactivated via surgical cuts (Figure 1A). As VSDI measure of neuronal activity, we used “region of interest” (ROI)-extracted fast, depolarization-mediated imaging signals (FDSs) (Figures 1A,B,D) (Tominaga et al., 2000; Airan et al., 2007; Refojo et al., 2011; von Wolff et al., 2011). Stimulus-evoked FDSs in hippocampal slice preparations reflect neuronal action potentials as well as excitatory postsynaptic potentials (EPSPs) and, therefore, are abolished or at least strongly diminished by voltage-gated Na^+^ channel blockers or ionotropic glutamate receptor antagonists and lowered extracellular Ca^2+^ concentrations, respectively (Tominaga et al., 2000; Airan et al., 2007; Carlson and Coulter, 2008; Chemla and Chavane, 2010; von Wolff et al., 2011).

### Theta-rhythmical EC/DG-input highly effectively generates neuronal activity flow through the hippocampus

First, we investigated whether theta-rhythmical (5 Hz) EC/DG-input triggers hippocampal network dynamics which, except for their rate of occurrence, differ from those elicited by non-theta (0.2 Hz) EC/DG-input (Figure 1C). Repetitive (0.2 Hz) EC/DG-input invariably caused prominent neuronal activity in the DG, only weak neuronal activity in the CA3 region, and no distinct neuronal activity in area CA1 (Figures 1D,E). In contrast, 5 Hz EC/DG-input reliably generated waves of neuronal activity which propagated through the entire trisynaptic circuit of the hippocampus (HTC) (Figures 1D–G). These “HTC-Waves” started to appear in an initially progressive manner approximately one second after the onset of the EC/DG-input and precisely followed the input rhythm (Figures 1D,E; **Supplementary Video**). Another observation was that neuronal activity in the DG markedly decreased during 5 Hz EC/DG-input (Figure 1E). This decremental activity possibly resulted from a known habituation process that involves GABAergic neurotransmission (Teyler and Alger, 1976). A pharmacological blockade of ionotropic glutamate receptors fully abolished HTC-Waves (Figure 1H).

The CA3 (stratum lucidum) field potential recordings suggest that HTC-Waves involve high-frequency firing of CA3 pyramidal neurons. This is deduced from multiple population spikes which accompanied the typically fast-rising mossy fiber field EPSP (fEPSP) (Toth et al., 2000; Nicoll and Schmitz, 2005) (Figure 1I). Both the rate and the number of these population spikes (173 ± 19 Hz and Figure 1I) correspond to the characteristics of physiological burst firing of CA3 pyramidal neurons (Andersen et al., 2007, p. 157). Population spikes were also interlaced in CA1 stratum radiatum (Schaffer collateral) fEPSPs (Figure 1I), indicating that HTC-Waves elicit action potentials in CA1 pyramidal neurons, which mediate hippocampal output to numerous brain structures, including the entorhinal cortex (Andersen et al., 2007).

We further tested whether non-theta (1 and 20 Hz) EC/DG-input generates activity propagations through the HTC. This was the case, but these activity propagations were considerably less strongly pronounced than those produced by 5 Hz EC/DG-input (Figure 1J). In addition, we found that the aforementioned decline of DG activity more heavily took place during 20 Hz EC/DG-input. The opposite scenario was observed for 1 Hz EC/DG-input (Figure 1K).

**Figure 2:**
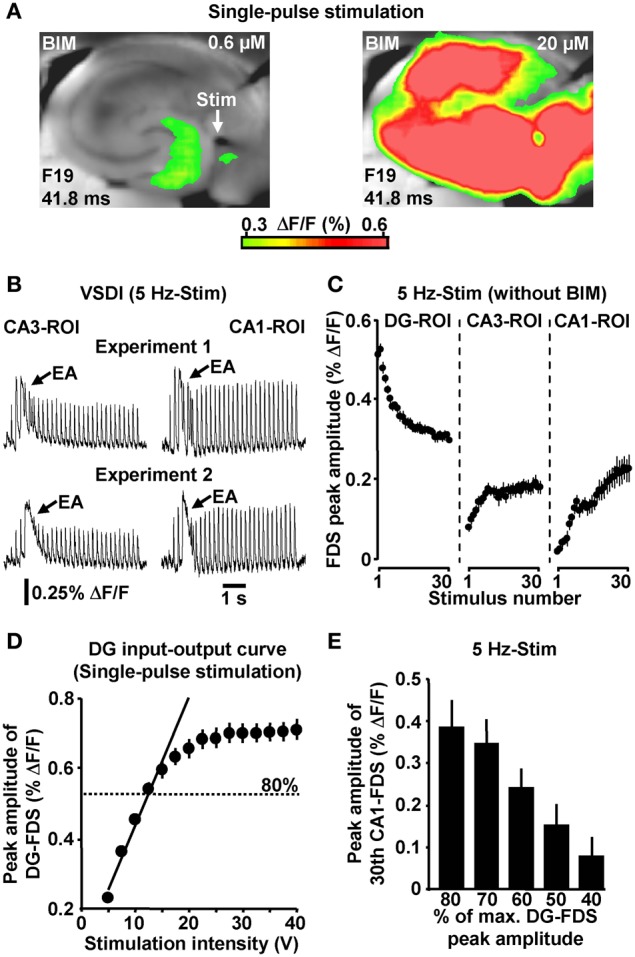
**(A)** Single-pulse evocation of EC/DG-input caused moderate, spatially restricted neuronal activity in the hippocampus in BIM (0.6 μM)-treated slices. In the same experiments, an increase in the concentration of BIM to 20 μM led to enormous (epileptiform) activity in all hippocampal subfields. **(B)** Examples of epileptiform activity (EA) induced by 5 Hz EC/DG-input. **(C)** 5 Hz EC/DG-input evoked HTC-Waves also in the absence of BIM (*N* = 10 slices/6 mice). **(D)** DG input–output curve obtained by single-pulse evocations of EC/DG-input (*N* = 8 slices/4 mice). **(E)** PP stimulation at intensities below of that producing DG-FDSs with a peak amplitude of approximately 80% of the highest attainable value also triggered HTC-Waves (*N* = 7 slices/5 mice).

If not stated otherwise, all brain slice experiments were carried out in the presence of 0.6 μM bicuculline methiodide (BIM). We used the GABA_A_ receptor antagonist BIM at this weakly blocking dose (Figure 2A) for the following reasons: first, compared to the relatively short axons of most hippocampal GABAergic interneurons (Andersen et al., 2007), projections from excitatory neurons are cut to a greater extent during preparation of slices. This probably leads to an artificial enhancement of inhibition in large-scale hippocampal circuits *in vitro* (e.g., Iijima et al., 1996). And second, the voltage-sensitive dye used (Di-4-ANEPPS) possibly slightly potentiated GABA_A_ receptor function (Mennerick et al., 2010). BIM almost never led to epileptiform activity in the hippocampal subfields under investigation. Nevertheless, we always paid particular attention to such activity and, in the very rare cases in which it was observed (<1% of experiments) (Figure 2B), slices were discarded from subsequent analysis. Although less strongly pronounced, HTC-Waves also reliably occurred in the absence of BIM (Figure 2C).

To ensure proper signal-to-noise ratios and minimize experimental variability in HTC dynamics, we evoked EC/DG-input using relatively high stimulation intensities. In detail, the intensity of voltage stimulation was adjusted in a manner to produce DG-FDSs with peak amplitudes of approximately 80% of the highest attainable value. These FDSs ranged at the end of the linear upturn of the respective input–output curve (Figure 2D). Hence, EC/DG-input probably caused spiking of numerous DG granule cells. Under *in vivo* conditions, however, these neurons have been found to be sparsely active (Jung and McNaughton, 1993; Leutgeb et al., 2007). We therefore addressed the question whether HTC-Waves likewise can occur if only a small number of trisynaptic interconnections becomes activated. Providing substantial support for this scenario, HTC-Waves were also detectable with considerably lower activity states of the DG and continuously declined, rather than abruptly dropped off in their strength if the stimulation intensity was significantly decreased in a gradual manner (Figure 2E).

### HTC-Waves *in vivo*

**Figure 3:**
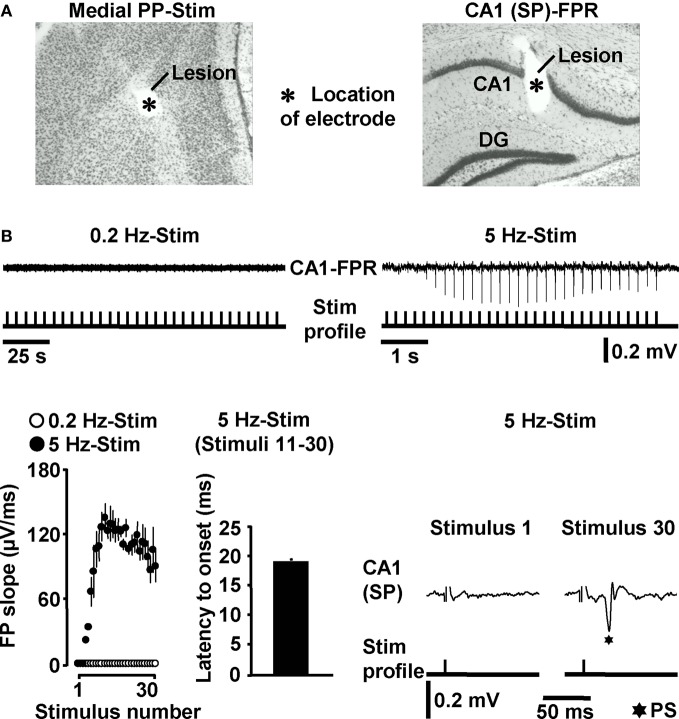
**HTC-Waves *in vivo*. (A)** Verification of electrode positions in one of the *in vivo* experiments (“SP” stands for “stratum pyramidale”). **(B)** Representative FPR traces and quantification and characteristics of neuronal activities recorded from four mice. Stimulus artifacts in FPR traces were truncated for clarity. 0.2 Hz PP stimulation was always conducted 5 min after 5 Hz PP activation.

Next, we addressed the question whether HTC-Waves also occur under *in vivo* conditions. For this purpose, we electrically stimulated the medial PP (Tang and Dani, 2009) in anesthetized mice and conducted field potential recordings in the CA1 stratum pyramidale (Figure 3A). A stepwise increase in the stimulation intensity (0.5 V steps) reliably exposed a threshold (3.4 ± 0.6 V, *N* = 4 mice) at which CA1 population spikes appeared during 5 Hz PP activation. These population spikes emerged in a nearly identical manner and with virtually equal latencies to onset (~18 ms) as the CA1 neuronal responses in the *in vitro* experiments. Again, this phenomenon could never be observed if the PP was stimulated at 0.2 Hz (Figure 3B). Only with higher stimulation intensities, the recordings displayed an additional potential deflection (i.e., a fEPSP) which, according to the latency to onset (~10 ms), most likely resulted from direct PP input to area CA3 (Andersen et al., 2007). A possible explanation for this connection is that, due to the distinct dendritic attenuation of direct PP input to CA3 pyramidal neurons (Urban et al., 1998), the concomitant fEPSP merely became detectable under our experimental settings if a relatively high number of CA3-targeting PP fibers was activated.

### HTC-Waves depend on frequency facilitation of mossy fiber to CA3 synaptic transmission

**Figure 4:**
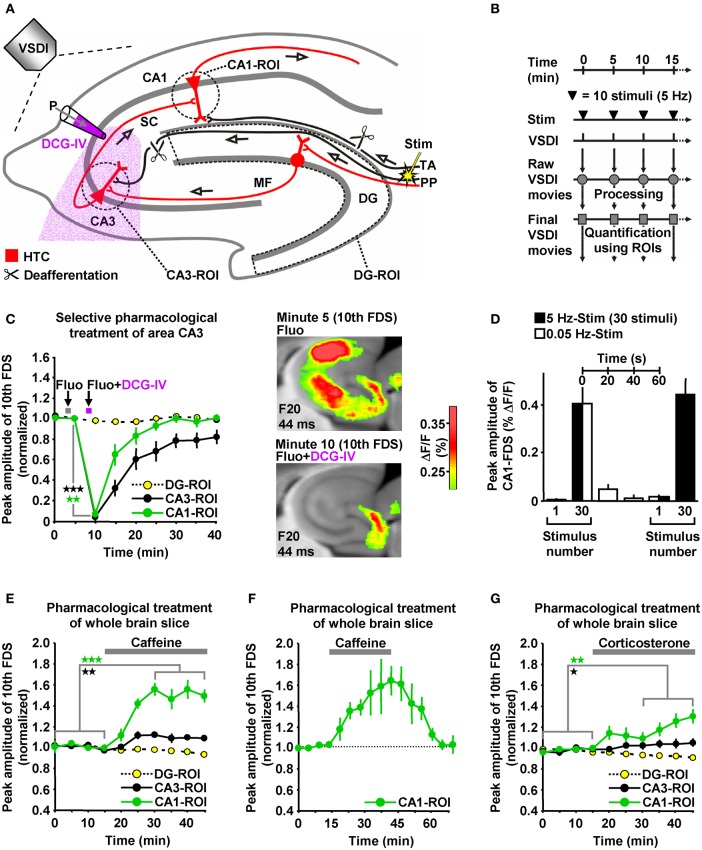
**HTC-Waves depend on frequency facilitation of mossy fiber to CA3 synaptic transmission and are rapidly boosted by caffeine and corticosterone. (A)** Experimental arrangement used for the investigations shown in **(C–G)** (“P” stands for “pressure”). **(B)** Experimental protocol used for the investigations shown in **(C** and **E–G)**. **(C)** A selective shutdown of mossy fiber synaptic transmission by a fluorescein (Fluo; 10 μM)-guided administration of the mGluR2 agonist DCG-IV (30 μM; 1 min) specifically to area CA3 abolished HTC-Waves without affecting DG activity (*N* = 8 slices/6 mice). **(D)** During low-frequency (0.05 Hz) EC/DG-input following 5 Hz EC/DG-input, the HTC reverted to its locked state regarding passages of neuronal activity (*N* = 6 slices/3 mice). **(E,F)** Caffeine (5 μM) quickly boosted HTC-Waves in a reversible manner (**E**, *N* = 8 slices/4 mice; **F**, *N* = 4 slices/3 mice). **(G)** Bath application of 100 nM corticosterone to slices rapidly amplified HTC-Waves (*N* = 7 slices/4 mice). **(C,E,G)** *p < 0.05 (paired *t*-test); **p < 0.01 (paired *t*-test); ***p < 0.001 (paired *t*-test).

The delayed and initially progressive appearance of HTC-Waves (Figures 1D,E; **Supplementary Video**) indicates that the 5 Hz EC/DG-input somehow unlocked a “gate” in the HTC for passages of neuronal activity. We considered frequency facilitation of mossy fiber to CA3 synaptic transmission (Toth et al., 2000; Nicoll and Schmitz, 2005) as the pivotal unlocking mechanism. Corroborating this notion, such frequency facilitation strongly took place during 5 Hz EC/DG-input, while neuronal activity in the DG decreased (Figures 1D,E,I). HTC-Waves and CA3-FDSs disappeared if glutamate release at CA3 mossy fiber terminals was selectively blocked by applying the mGluR2 agonist DCG-IV (Toth et al., 2000; Nicoll and Schmitz, 2005) specifically to area CA3 (Figures 4A–C).

Frequency facilitation at mossy fiber synapses is a short-lasting form of synaptic plasticity (Toth et al., 2000). We therefore postulated that HTC-Waves dissipate within a couple of seconds if 5 Hz EC/DG-input is followed by low-frequency (0.05 Hz) EC/DG-input (Toth et al., 2000). This was the case and reoccurring 5 Hz EC/DG-input again generated HTC-Waves which displayed a delayed and initially progressive emergence (Figure 4D).

### HTC-Waves are rapidly boosted by the cognitive enhancer caffeine and the stress hormone corticosterone

HTC-Waves might provide a valuable assay for studying drug effects on polysynaptic activity flow through the hippocampus. For this reason and due to the fact that the hippocampus plays a fundamental role in cognitive processes, we examined the impact of the cognitive enhancer caffeine (Nehlig, 2010) on HTC-Waves. As already suggested by the DCG-IV experiments (Figure 4C), stimulation trains containing 10 stimuli allowed for stable baseline recordings of HTC-Waves (Figures 4B,E). Subsequent bath application of caffeine at a concentration found in the cerebrospinal fluid of humans after intake of 1–2 cups of coffee [5 μM (Fredholm et al., 1999)] boosted HTC-Waves (Figure 4E).

It has been shown that caffeine can mediate a presynaptic form of LTP at CA3-CA1 synapses (Martín and Buno, 2003). On this account, we additionally performed wash-out experiments to test whether such LTP might contribute to the caffeine-induced enhancement of HTC-Waves. Contradicting this possibility, CA1-FDSs reverted to baseline strength if caffeine was removed from the superfusion medium (Figure 4F).

To also investigate potential effects of a natural neuromodulator on HTC-Waves, we administered the stress hormone corticosterone to slices. Corticosterone was chosen since the rodent hippocampus is a target of this glucocorticoid (McEwen, 1999; Andersen et al., 2007), but experimental demonstrations of how it affects neuronal activity flow through the HTC network are lacking to date. As observed for caffeine, corticosterone (100 nM) rapidly amplified HTC-Waves (Figure 4G).

### HTC-Waves evoke NMDA receptor-dependent CA1 LTP

**Figure 5:**
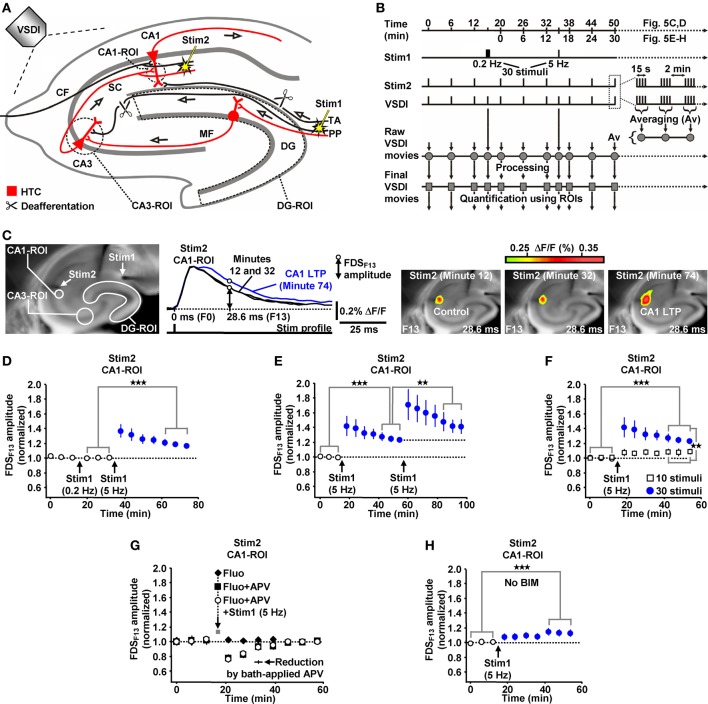
**HTC-Waves evoke NMDA receptor-dependent CA1 LTP. (A,B)** Experimental arrangement and protocol used for the investigations shown in **(C–H)** (“CF” in **A** stands for “commissural fiber”). **(C)** Illustration and outcome of one of the experiments summarized in **(D)**. **(D)** 5 Hz EC/DG-input for 6 s elicited CA1 LTP, which was not saturated as shown in **(E)** (**D**, *N* = 13 slices/8 mice; **E**, *N* = 8 slices/5 mice). **(F)** 5 Hz EC/DG-input for 2 s failed to evoke statistically significant CA1 LTP [open squares; *N* = 7 slices/5 mice; *p* > 0.05 (paired *t*-test)]. Blue circles represent data shown in **(E)**. **(G)** A blockade of NMDA receptors at CA3-CA1 synapses by a Fluo (10 μM)-guided administration of APV (200 μM; 1 min) specifically to area CA1 prevented the formation of CA1 LTP [*N* = 6 slices/4 mice; *N* = 5 slices/4 mice for control experiments with Fluo; *N* = 6 slices/4 mice for control experiments with Fluo + APV; and *N* = 5 slices/3 mice for control experiments with APV (50 μM) bath-applied to slices (for rationale see “Materials and Methods”)]. **(H)** 5 Hz EC/DG-input evoked CA1 LTP also in the absence of BIM (*N* = 10 slices/6 mice). **(D–F,H)** **p < 0.01 (paired or unpaired *t*-test); ***p < 0.001 (paired *t*-test).

**Figure 6:**
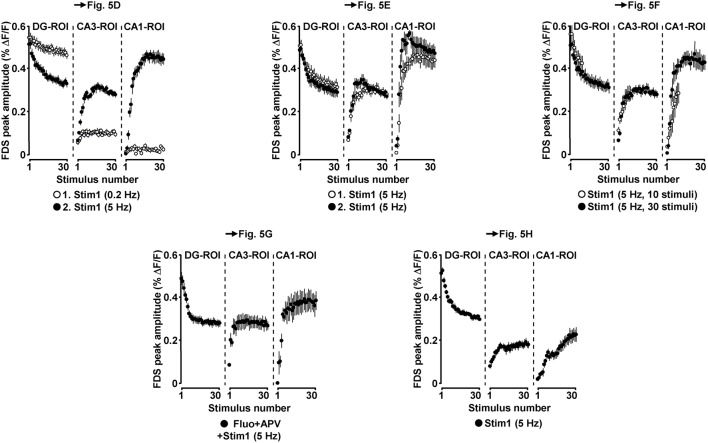
**Neuronal activities in hippocampal subregions triggered by EC/DG-input in the LTP experiments depicted in Figure 5**.

Due to the inherent high-frequency discharges of CA3 pyramidal neurons (Figure 1I), we expected HTC-Waves to yield a kind of “theta-burst” activation of CA3-CA1 synapses. Theta-burst stimulation of CA3-CA1 projections in turn is known to effectively induce CA1 LTP (Bliss and Collingridge, 1993). Therefore, we finally tested whether 5 Hz EC/DG-input can elicit CA1 LTP. To enable the imaging of the putative formation of CA1 LTP, we refined our VSDI assay. To accurately gauge the strength of neurotransmission at CA3-CA1 synapses, we repeatedly activated them by directly stimulating the Schaffer collateral-commissural pathway at a low frequency (Bliss and Collingridge, 1993) (Figures 5A–C). Owing to methodological reasons (see “Materials and Methods”), we employed the amplitude of the resultant CA1 (stratum radiatum)-FDSs at imaging frame 13 (28.6 ms) after the stimulation pulse (FDS_F13_ amplitude) as measure of changes in the strength of neurotransmission at CA3-CA1 synapses (Figure 5C; “Materials and Methods”). As confirmed by field potential recordings performed in the CA1 stratum radiatum, this analysis showed that 5 Hz EC/DG-input for 6 s was sufficient to evoke CA1 LTP (Figures 5C,D; “Materials and Methods”). The CA1 LTP could be increased in its magnitude by a further train of HTC-Waves (Figure 5E). CA1 LTP critically depended on the number of HTC-Waves (Figure 5F) and the activation of NMDA receptors (Bliss and Collingridge, 1993) as revealed by the administration of the NMDA receptor blocker APV specifically to area CA1 (Figure 5G). 5 Hz EC/DG-input for 6 s also elicited CA1 LTP in the absence of BIM (Figure 5H). In all LTP experiments, we monitored hippocampal activity resulting from EC/DG-input by means of VSDI (Figure 6).

## Materials and methods

### Animals

For all experiments, adult (8–12 week-old) male C57BL/6N mice were used. All experimental procedures were approved by the Committee on Animal Health and Care of the local governmental body and performed in strict compliance with the guidelines for the care and use of laboratory animals set by the European Community.

### Preparation and staining of brain slices

Mice were anesthetized with isoflurane and decapitated. All following steps were done in ice-cold sucrose-based saline (Bischofberger et al., 2006; Refojo et al., 2011; von Wolff et al., 2011) saturated with carbogen gas (95% O_2_/5% CO_2_). This saline (pH 7.4) consisted of (in mM): 87 NaCl, 2.5 KCl, 25 NaHCO_3_, 1.25 NaH_2_PO_4_, 0.5 CaCl_2_, 7 MgCl_2_, 25 glucose, and 75 sucrose. After decapitation, the brain was rapidly removed from the cranial cavity and separated into its hemispheres. Next, the hemispheres were prepared for the slicing procedure by a special transversal cut, which is sometimes called “magic cut” (Bischofberger et al., 2006). We cut the hemispheres at angles optimized to conserve the intrahippocampal axonal projections along the DG-CA axis as best as possible (Bischofberger et al., 2006; Refojo et al., 2011; von Wolff et al., 2011). Subsequently, 350 μm-thick horizontal slices containing the hippocampus were cut using a vibratome (HM650V; Thermo Scientific).

After preparation, slices were incubated in carbogenated sucrose-based saline for 30 min at 34°C. Subsequent staining of slices with the voltage-sensitive dye Di-4-ANEPPS (Tominaga et al., 2000; Airan et al., 2007; Refojo et al., 2011; von Wolff et al., 2011) (dissolved in DMSO to a 20.8 mM stock solution) was carried out at room temperature (23–25°C). For staining, slices were kept for 15 min in carbogenated physiological saline containing Di-4-ANEPPS [7.5 μg/ml; <0.1% DMSO (Refojo et al., 2011; von Wolff et al., 2011)]. The physiological saline (pH 7.4) consisted of (in mM): 125 NaCl, 2.5 KCl, 25 NaHCO_3_, 1.25 NaH_2_PO_4_, 2 CaCl_2_, 1 MgCl_2_, and 25 glucose. Afterwards, slices were stored at room temperature for at least 30 min in Di-4-ANEPPS-free, but BIM (0.6 μM)-containing carbogenated physiological saline.

### Brain slice experiments

All brain slice experiments were carried out at room temperature. In the recording chamber, slices were fixed with a platinum frame/nylon string harp and continuously superfused with BIM (0.6 μM)-containing carbogenated physiological saline (4–5 ml/min flow rate). Pilot experiments revealed that HTC-Waves can be reliably evoked in slices that were cut at a distance between 2.3 and 3.0 mm (±0.1 mm) from the ventral base of the brain. For this reason, all *in vitro* investigations were performed in these particular slices. Accordingly, experiments were conducted in the dorsal hippocampus or in the transition zone from the dorsal to the ventral hippocampus (Maggio and Segal, 2007; Fanselow and Dong, 2010).

**Figure 7:**
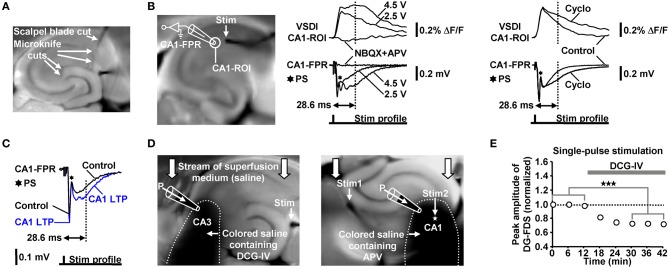
**(A)** Illustration of the deafferentations performed in hippocampal slices. **(B)** Characterization of the FDS_F13_ amplitude by simultaneously recorded CA1-FDSs and CA1 (stratum radiatum) fEPSPs. Cyclothiazide (Cyclo), NBQX, and APV were bath-applied at concentrations of 100, 5, and 50 μM, respectively. **(C)** 5 Hz EC/DG-input for 6 s caused a long-lasting increase in the slope and the amplitude of CA1 (stratum radiatum) fEPSPs 28.6 ms after the stimulation pulse. The field potential depicting the CA1 LTP was recorded 40 min after the 5 Hz EC/DG-input. **(D)** Illustration of the Fluo-guided administration of DCG-IV and APV to areas CA3 and CA1, respectively. For a better image presentation of the spread of the pressure-ejected saline, Ponceau S was added instead of Fluo to the pipette solution. **(E)** Bath application of 1 μM DCG-IV to slices reduced neuronal activity in the DG (*N* = 6 slices/3 mice). ***p < 0.001 (paired *t*-test). **(B,C)** Stimulus artifacts in FPR traces were truncated for clarity.

We evoked EC/DG-input by electrical stimulation of the PP, which also contains fibers that directly innervate CA3 pyramidal neurons (Andersen et al., 2007). We cut these fibers at the point where they exit the DG. Temporoammonic projections (Andersen et al., 2007) were likewise functionally inactivated. The deafferentations were accomplished by means of a tapered scalpel blade and a custom-made “microknife” (approximately 100 μm blade length) (Figure 7A).

### VSDI

VSDI and data analysis were performed using the MiCAM02 hard- and software package (BrainVision) (Refojo et al., 2011; von Wolff et al., 2011). The tandem-lens fluorescence microscope was equipped with the MiCAM02-HR camera and the 2× and 1× lens at the objective and condensing side, respectively (for further technical details see http://www.scimedia.com). Acquisition settings were as follows: 88 × 60 pixels frame size, 36.4 × 40.0 μm pixel size, and 2.2 ms sampling time.

### Processing and quantification of VSDI data

From recorded VSDI signals, the fractional change in fluorescence (Δ*F/F*) was calculated. For all quantifications, Δ *F/F* values were spatially and temporally smoothed using a 3 × 3 × 3 average filter. VSDI signals presented in images and the video were smoothed with a 5 × 5 × 3 average filter. To attenuate slow signal components produced from bleaching of the voltage-sensitive dye (Carlson and Coulter, 2008) and slight summation of 5 and 20 Hz neuronal responses, we afterwards applied a weak high-pass filter of the MiCAM02 software (τ = 220 ms) to the imaging data. Pixelation of images and the video was reduced by the interpolation function of the MiCAM02 software.

For analysis of neuronal population activity in hippocampal subregions, three standardized ROIs were manually set according to anatomical landmarks. The circular CA3-ROI (*r* = 4 pixels) was positioned into the CA3 region near the DG, but not overlapping with it. The circular CA1-ROI (*r* = 4 pixels) was placed into the CA1 subfield with a distance of approximately 200 μm from the visually identified distal end of the stratum lucidum. The CA3-ROI spanned the stratum oriens, stratum pyramidale, and stratum lucidum/radiatum. This was also the case for the CA1-ROI, with the exception of the LTP experiments where it only covered the stratum radiatum (*r* = 2 pixels). The DG-ROI enclosed the fascia dentata (without the hilus) and was created by the polygon-drawing function of the MiCAM02 software. The average of smoothed Δ *F/F* values within a particular ROI served as final measure of neuronal population activity.

### Electrophysiology *in vitro*

Field potentials in the CA3 stratum lucidum and CA1 stratum radiatum were recorded using glass microelectrodes (1 MΩ open-tip resistance) that were filled with BIM (0.6 μM)-containing physiological saline. Recording data were low-pass filtered at 1 kHz and digitized at 5 kHz.

### Electrophysiology *in vivo*

All *in vivo* recordings were performed in the left dorsal hippocampus and carried out under urethane anesthesia (750 mg/kg body weight; i.p.). An additional dose of urethane (250 mg/kg) was added 30–60 min later (Irikura et al., 1995). The temperature of the anesthetized animal was constantly controlled with a rectal probe and maintained at 36°C with a heating pad (homeothermic blanket system; Harvard Apparatus). During preparation of the skull and the recording session, the animal was permanently fixed in a computer-guided stereotactic device (Angle Two™; myNeurolab). The coordinates for electrical stimulation and recording of neuronal activity were as follows: medial PP (stimulation): 0.2 mm posterior and 2.9 mm lateral from lambda, 1.3 mm ventral from brain surface (Tang and Dani, 2009); CA1 stratum pyramidale (recording): 2.2 mm posterior and 1.2 mm lateral from bregma, 1.3 mm ventral from brain surface (Franklin and Paxinos, 1997). For recording, tungsten microelectrodes (TM33A20; World Precision Instruments) were used. A gold wire with a ball-shaped ending served as the indifferent electrode and was placed epidurally through a small hole in the skull (250 μm drilling diameter) at the rostral part of the right os frontale. Recording data were band-pass filtered (2 Hz cutoff frequency for high-pass filter and 1 kHz for low-pass filter) and digitized at 5 kHz. After termination of the experiment, electrical lesions were set at stimulation and recording sites (3 μA for 3 min; negative polarity). Each brain was then cut into 30 μm sections and stained (standard cresyl violet staining) to verify the position of each electrode.

### Electrical stimulation techniques

In all experiments, neuronal activity was evoked by square pulse electrical stimuli (200 μs pulse width). For stimulation of the medial PP *in vivo*, custom-made monopolar tungsten electrodes (Teflon-insulated to the tip of 75 μm diameter) were used.

For electrical stimulation *in vitro*, we also used custom-made monopolar tungsten electrodes (Teflon-insulated to the tip of 50 μm diameter). These electrodes allowed for a very precise placement into the neuronal tissue and did not interfere with VSDI (e.g., by producing irritating shadows as observed with other electrodes). A highly localized electrical stimulation was achieved by positioning the indifferent electrode far away from the slice in the recording chamber. In LTP experiments, in which two stimulation electrodes were employed, the currently unused electrode was uncoupled from the stimulation device. This was done to prevent an indirect application of voltage to the inactive electrode.

The electrode employed for evocation of EC/DG-input was placed on the visually identified PP near its entry zone to the DG. Direct stimulation of the Schaffer collateral-commissural pathway in LTP experiments was performed near the CA3 averted boundary of the CA1-ROI, which was used to monitor putative alterations in the strength of neurotransmission at CA3-CA1 synapses. This was done to antidromically (Bliss and Collingridge, 1993) activate a large fraction of the CA3-CA1 synapses that were also activated by the later induced HTC-Waves. Thereby, commissural fibers and Schaffer collaterals which were cut somewhere in the CA3 or CA1 region during preparation of slices became also recruited. To minimize the probability that neuronal activity within the CA1-ROI overly resulted from activation of such “irrelevant” fibers, we used a stimulation intensity which produced a strong fEPSP and, thus, a concomitant population spike (Figures 7B,C).

In contrast to field potential recordings, VSDI displays action potentials and EPSPs always as signals exhibiting the same deflection course (Carlson and Coulter, 2008). Therefore, neither the peak amplitude nor the slope of CA1-FDSs could be used as proper measure of changes in the strength of neurotransmission at CA3-CA1 synapses. To overcome this problem and thereby become also able to conduct the LTP experiments via VSDI, we employed the amplitude of a later CA1-FDS component as such a measure. Several sets of pilot experiments identified the amplitude of CA1-FDSs at imaging frame 13 (28.6 ms) after the stimulation pulse (FDS_F13_ amplitude) to be suited. As shown by simultaneous field potential recordings, the FDS_F13_ amplitude reflects the amplitude of the glutamatergic fEPSP at a certain time point of its decaying phase. At this time point, the fEPSPs did not anymore mingle with action potentials fired by CA1 pyramidal neurons (Figure 7B). The intensity of voltage stimulation was adjusted in a manner to produce a FDS_F13_ amplitude with a value in the range between 60–80% of the peak amplitude of the CA1-FDS (Figures 5C, 7B). If performed in this way, the FDS_F13_ amplitude could be increased by enhancing the stimulation intensity. An increase in the FDS_F13_ amplitude was also seen after adding the AMPA receptor potentiator cyclothiazide at a concentration of 100 μM (Eder et al., 2003) to the superfusion medium. The same effects could be observed in the field potential recordings (Figure 7B). Additionally vindicating the use of the FDS_F13_ amplitude in the LTP experiments, 5 Hz EC/DG-input for 6 s, which evoked CA1 LTP as measured with VSDI, caused a long-lasting increase in the slope and the amplitude of fEPSPs 28.6 ms after the stimulation pulse (Figure 7C).

### Fluorescein (Fluo)-guided administration of drugs to brain slices

For a non-invasive pressure application of a given drug to a defined hippocampal subfield, we positioned a glass microelectrode (0.2–0.3 MΩ open-tip resistance) within a certain region close to the surface of the slice under investigation. The electrode was filled with physiological saline containing BIM (0.6 μM), Fluo (10 μM), and the drug to be applied. Fluo, whose fluorescence was excited by means of light emitted from a UV diode, served to visually monitor the spread of the pressure-ejected fluid via a binocular. A spatially restricted spread was achieved by capitalizing on the stream of the superfusion medium through the recording chamber, by appropriately adjusting the strength of the pressure applied to the electrode, and by a specific orientation of the slice within the recording chamber (Figure 7D). Since the drugs (DCG-IV and APV) had to rapidly penetrate the neuronal tissue by diffusion, we used somewhat higher concentrations of them than typically bath-applied to reach a maximal pharmacological effect. In all sets of experiments, control applications of Fluo alone were carried out. To verify the spatial specificity of the Fluo-guided drug administration technique, we bath-applied DCG-IV at a concentration of 1 μM (Kew et al., 2002; Nicoll and Schmitz, 2005) to slices. Consistent with its inhibitory effect on glutamatergic neurotransmission at PP synapses (Kew et al., 2002), DCG-IV decreased neuronal activity in the DG (Figure 7E). This effect was not observed if DCG-IV was applied specifically to area CA3 (Figure 4C). We furthermore bath-applied APV at a concentration of 50 μM to slices. This was done to determine the concentration of pressure-applied APV that leads to a complete block of NMDA receptors. Bath-applied APV reduced the FDS_F13_ amplitude of CA1-FDSs almost to the same extent as 200 μM APV that was pressure-applied to the CA1 subfield for 1 min (Figure 5G).

### Chemicals

BIM, caffeine, corticosterone, Di-4-ANEPPS, Fluo, Ponceau S, urethane, and all salts for the saline solutions were purchased from Sigma-Aldrich. APV and NBQX were from Ascent Scientific, cyclothiazide and DCG-IV from Tocris, and isoflurane from Abbott. Corticosterone was dissolved in ethanol. During experimentation (including baseline recording), ethanol was present at a concentration of 0.009%.

### Statistics

Two-tailed *t*-tests were used for statistical analyses and run in SigmaStat (Systat Software), with significance declared at *p* < 0.05. Data are given as mean ± s.e.m.

## Discussion

In the present study, we directly examined whether theta-rhythmical synaptic input from the entorhinal cortex to the dentate gyrus (EC/DG-input) is able to evoke hippocampal network dynamics which can elicit CA1 LTP. For this purpose, we established a VSDI assay in mouse brain slices allowing the investigation of manifold aspects of polysynaptic activity flow within the hippocampus. By means of this assay and complementary *in vivo* recordings, we show that 5 Hz EC/DG-input (resulting from synchronous 5 Hz spike trains in PP fibers) highly effectively generates waves of neuronal activity which propagate through the entire trisynaptic circuit of the hippocampus. These HTC-Waves precisely follow the rhythm of the EC/DG-input, involve high-frequency firing (>100 Hz) of CA3 pyramidal neurons, and induce NMDA receptor-dependent CA1 LTP within a few seconds.

As evident from recently published data, EC theta oscillations entail sequences of synchronous theta-rhythmical spiking in layer II stellate cell ensembles (Mizuseki et al., 2009; Quilichini et al., 2010; Burgalossi et al., 2011). Such coordinated activity in neuronal populations of the medial temporal lobe can be crucial for the formation of explicit memories (Rutishauser et al., 2010). Furthermore, *in vivo* recordings corroborate that EC theta oscillations are tightly associated with theta-frequency discharges in DG granule cells (Jung and McNaughton, 1993; Skaggs et al., 1996; Mizuseki et al., 2009). The resultant frequency facilitation at mossy fiber synapses onto CA3 pyramidal neurons is presumably such strongly pronounced that even unitary EPSPs are able to fire these cells (Jonas et al., 1993; Toth et al., 2000). Frequency depression at mossy fiber synapses onto CA3 inhibitory interneurons (Toth et al., 2000) might indirectly promote action potential generation in CA3 pyramidal cells. These neurons typically respond with burst spiking (100–300 Hz) to suprathreshold depolarizations (Andersen et al., 2007 p. 157). Pharmacologically induced burst discharges of CA3 pyramidal cells as well as theta-burst stimulation of CA3-CA1 projections efficiently evoke CA1 LTP (Buzsáki et al., 1987; Bliss and Collingridge, 1993). From all this, it is very likely that HTC-Waves and induction of CA1 LTP by them also naturally take place at the level of sparse numbers (Jung and McNaughton, 1993; Whitlock et al., 2006; Leutgeb et al., 2007) of trisynaptic interconnections and the appendant microcircuits [e.g., associational loops (Andersen et al., 2007)]. Such a scenario does not exclude contributions of other afferent inputs [e.g., direct EC input to area CA3 (Andersen et al., 2007)] to CA1 LTP formation, but ascribes a major role to EC/DG-input. This can explain why some forms of hippocampus-dependent learning require the integrity of the full HTC (Nakashiba et al., 2008) and become impaired if mossy fiber to CA3 synaptic transmission is pharmacologically suppressed (Daumas et al., 2009).

We show that the occurrence and strength of activity propagations through the HTC critically depends on the frequency and persistency of EC/DG-input (Figures 1E,J). It is tempting to speculate that this connection reflects a basic filter mechanism of the hippocampus regarding EC inputs that, as suggested by one of our data sets, is modifiable by the attention-enhancing drug caffeine (Nehlig, 2010). In addition, we observed that DG-FDSs increasingly decline in their magnitude during 0.2, 1, 5, and 20 Hz EC/DG-input, respectively (Figure 1K). In combination with the fact that frequency facilitation at mossy fiber synapses develops stronger with higher frequencies (Toth et al., 2000), it is thus possible that the DG-CA3 complex acts as a “low-order band-pass filter,” wherein the DG circuitry serves as the “low-pass unit” and the CA3 mossy fiber system as the “high-pass device.” If so, this filter would be effectually passed by incoming information encoded in the theta-frequency range. Hence, an important physiological function of theta-rhythmical spiking of EC stellate cells might be to drive sensory information through the whole entorhinal-hippocampal loop. In line with this hypothesis, Iijima et al. (1996) demonstrated that pharmacologically induced reverberating activity within the entorhinal cortex (close to the theta-frequency range) causes invasion of activity into the hippocampus upon several reverberations.

We found that activity propagations through the HTC as well occur during 1 and 20 Hz EC/DG-input. It is therefore likely that also such EC/DG-input can elicit CA1 LTP. However, the markedly higher strength of HTC-Waves (Figure 1J), together with the results from a previous study (Capocchi et al., 1992), argues for a more effective induction of CA1 LTP by theta-rhythmical EC/DG-input. Capocchi and colleagues show that theta-burst stimulation of CA3-CA1 projections leads to considerably stronger CA1 LTP than 1 and 20 Hz burst stimulation.

It is important to mention that “polysynaptic LTP” in the CA1 subfield has already been described by Buzsáki (1988) and Nakagami et al. (1997). However, there are significant differences to our work. In the first study, a long-lasting enhancement of CA1 population spikes was induced by several high-frequency (400 Hz) trains of stimuli delivered to the angular bundle. With regard to the available literature, it is questionable if 400 Hz spiking represents a physiological activity pattern of EC stellate cells and other EC neurons that send projections via the PP to the hippocampus (e.g., Andersen et al., 2007; Mizuseki et al., 2009; Quilichini et al., 2010; Burgalossi et al., 2011). Moreover, Buzsáki states that direct EC inputs to areas CA3 and CA1, which we intentionally eliminated in our *in vitro* experiments, most likely played an essential role in the induction of the population spike LTP. Interestingly, he also points out that trisynaptic interconnections, which can be regarded as the main route of information flow through the hippocampus (Nicoll and Schmitz, 2005; Andersen et al., 2007; Neves et al., 2008), are not able to follow high-frequency PP activity. In the second study, Nakagami and colleagues conducted VSDI measurements in rat brain slices and report that a 1-s-long high-frequency (100 Hz) train of stimuli delivered to the dendritic field (molecular layer) of the DG caused “trisynaptic LTP induction.” Yet, it is safe to assume that the stimulation paradigm used did not only elicit neurotransmission at PP fiber synapses onto DG granule cells, but also led to direct PP input to CA3 pyramidal neurons and non-synaptic excitation of DG granule cells. Furthermore, the authors provide no clear indication for the occurrence of LTP at CA3-CA1 synapses, e.g., by showing an increase in CA1 responses which were evoked by stimulation of the Schaffer collateral-commissural pathway and/or by inhibiting the LTP observed via a blockade of NMDA receptors.

In addition to proving that HTC-Waves can elicit CA1 LTP, we validated these activity propagations to be a valuable tool for studying effects of drugs and natural neuromodulators on neural signal flow through the hippocampus. In particular, we demonstrate that caffeine at a concentration found in the cerebrospinal fluid of humans after intake of 1–2 cups of coffee [5 μM (Fredholm et al., 1999)] quickly boosts HTC-Waves. This effect, which might partly underlie the beneficial action of caffeine on cognitive processes (Nehlig, 2010), disappeared within approximately 15 min after removing the alkaloid from the bath solution, thus contradicting an involvement of a previously described caffeine (10 mM)-induced form of CA1 LTP (Martín and Buno, 2003). We suppose that the enhancement of HTC-Waves resulted, at least in part, from the antagonistic action of caffeine on adenosine A_1_ receptors that has been reported to strengthen neurotransmission at CA3 mossy fiber and CA2 Schaffer collateral synapses (Kukley et al., 2004; Simons et al., 2012). We further demonstrate that the stress hormone corticosterone (100 nM) quite rapidly amplifies HTC-Waves. This phenomenon cannot be explained by genomic effects of corticosterone on neuronal activity (Groeneweg et al., 2011). However, it becomes increasingly evident that corticosterone also affects hippocampal functioning in a fast, non-genomic manner (Groeneweg et al., 2011; Popoli et al., 2012). Our data give support for such a modulatory action at the neuronal network level and indicate that corticosterone is able to expeditiously increase hippocampal output. It is conceivable that corticosterone, in this way, mediates fast negative feedback on the hypothalamic-pituitary-adrenal (HPA) axis (Holsboer, 2000). At the moment, we cannot rule out that facilitation of CA1 LTP formation (Wiegert et al., 2006) played a causal role in the corticosterone effect observed. Yet, its fast onset under moderate PP stimulation (Figure 4G) suggests the contribution of a long-term plasticity-independent change in glutamatergic neurotransmission, e.g., a mineralocorticoid receptor-mediated increase in glutamate-release probability (Karst et al., 2005).

To conclude, our work provides the first experimental evidence that synchronous theta-rhythmical spiking of EC stellate cells [including grid cells (Hafting et al., 2008; Burgalossi et al., 2011)] has the capacity to drive induction of CA1 LTP via the hippocampal trisynaptic pathway. HTC-Waves could be of use for studying whether alterations in cognitive abilities are linked to changes in neuronal activity flow through the hippocampus.

### Conflict of interest statement

The authors declare that the research was conducted in the absence of any commercial or financial relationships that could be construed as a potential conflict of interest.
